# Localization-guided segmentation of Gliomas via 3D ReFusionNet

**DOI:** 10.3389/fonc.2026.1832485

**Published:** 2026-09-23

**Authors:** Ling Chen, Jinzhu Chang, Qi Zhang, Fang Ren, Jianjun Song, Jianzhou Yang, Xiangyu Guo

**Affiliations:** 1 Department of Radiology, Heping Hospital Affiliated to Changzhi Medical College, Changzhi, China; 2 Department of Anesthesiology, Heping Hospital Affiliated to Changzhi Medical College, Changzhi, China; 3 Department of Public Health and Preventive Medicine, Changzhi Medical College, Changzhi, China

**Keywords:** brain tumor segmentation, MRI, localization module, mask module, localization-guided segmentation

## Abstract

**Purpose:**

This study presents a localization-guided two-stage 3D framework for the automated segmentation of brain tumors.

**Methods:**

ReFusionNet first estimates a single region of interest containing the tumor-bearing region and then segments the whole tumor, tumor core, and enhancing tumor within the localized volume. The framework was evaluated on the UPENN-GBM and BraTS 2021 datasets using recall, precision, Dice score, and the 95th-percentile Hausdorff distance (HD95).

**Results:**

On the UPENN-GBM test set, ReFusionNet-A achieved Dice scores of 0.90, 0.81, and 0.77 for the whole tumor, tumor core, and enhancing tumor, respectively. Its corresponding HD95 values were 11.68, 21.53, and 10.49. The localization-guided models generally showed higher precision and lower HD95 values for selected regions, but lower recall and Dice scores occurred in some tumor-core and enhancing-tumor comparisons. On BraTS 2021, ReFusionNet-C achieved competitive Dice scores, although its HD95 values were not the lowest among the models compared.

**Conclusion:**

ReFusionNet achieved competitive segmentation performance on the evaluated public datasets. Its principal observed advantages were higher precision and lower HD95 values in selected tumor regions, while performance gains were not uniform across all metrics.

## Introduction

Brain is the most complex organ of the human, controlling most of the activities of the body and consisting of many different types of cells. According to the GBD study, the incidence of CNS cancer has risen globally, leading not only to higher rates of mortality but also to significant disabling effects on patients (1). Among CNS cancers, the most common type is glioma, typically originating from glial cells and surrounding tissue lesions, with varying degrees of invasiveness and destructiveness (2). Gliomas have an irregular shape and ambiguous boundaries, which are the most challenging tumors to detect. Therefore, automatic brain tumor segmentation is valuable for assessing tumor characteristics and assisting doctors in diagnosis, treatment and survival prediction in clinical practice (3).

Magnetic resonance imaging (MRI) is a commonly used technology in the field of brain tumor detection. Compared to regular X-rays and CT scans, MRI offers high contrast for soft tissues without involving radiation or invasive procedures, making it the preferred imaging method when frequent scans are needed for diagnosis or therapy, especially in brain imaging (4). Sub-region segmentation of brain tumors using MRI plays a crucial role in helping doctors more accurately assess tumor characteristics, develop personalized treatment plans, and predict patient prognosis. Manual segmentation and automated segmentation are two common methods in medical image analysis. Manual and automated segmentation are two common methods in medical image analysis (5). Manual segmentation, typically performed by experienced doctors, produces highly accurate results and is still regarded as the ‘gold standard’ in the field. However, it heavily depends on the doctor’s expertise and clinical experience. Automated segmentation can process large volumes of data, greatly improving efficiency while avoiding the influence of personal factors, leading to more consistent outcomes (6). Combining both methods offers a balance between efficiency and accuracy.

Various techniques are available for automated tumor segmentation, ranging from traditional methods to deep learning-based approaches. Traditional methods often focus on identifying color, texture, or brightness characteristics of different image regions. These include techniques such as thresholding, region-based segmentation, edge-based segmentation, and clustering (7–10). With the advancement of computational resources, deep learning-based methods have emerged as the preferred choice for performing segmentation tasks reliably. These deep learning models include generative adversarial networks (GANs), recurrent neural networks (RNNs), and convolutional neural networks (CNNs), with CNNs remaining the most widely used method for brain tumor segmentation (11–13). FCN, U-Net, and SegNet are commonly used network architectures for semantic segmentation, achieving excellent segmentation performance in medical image analysis (14–16).

Existing two-stage segmentation approaches commonly use a coarse segmentation network to identify a region of interest, followed by a second network for refinement (17). However, direct application of conventional object-detection pipelines to brain tumor subregion segmentation is not straightforward. Unlike separate objects in natural images, the whole tumor, tumor core, and enhancing tumor regions are spatially nested and share irregular and indistinct boundaries (18, 19). Generating multiple region proposals for these subregions may therefore introduce redundant candidates and does not reflect their anatomical relationship.

To address this issue, we propose ReFusionNet, a localization-guided two-stage 3D segmentation framework. Instead of generating multiple object-level proposals, ReFusionNet initializes a single proposal from the complete MRI volume and uses a 3D localization network to estimate six spatial parameters that adapt its position and extent to each subject. The resulting proposal is designed to encompass the entire tumor-bearing region, which is subsequently transformed into a standardized three-dimensional region of interest for subregion segmentation. This design focuses the mask network on the tumor vicinity while preserving the nested relationship among the whole tumor, tumor core, and enhancing tumor regions.

Within the localized region, ReFusionNet employs a shared encoder and two complementary decoder pathways derived from U-Net and SegNet. Their outputs are integrated using additive, elementwise multiplicative, or concatenation-based fusion, resulting in the ReFusionNet-A, ReFusionNet-M, and ReFusionNet-C variants, respectively. The principal contributions of this study are as follows:

1. We introduce a single-proposal localization strategy adapted to the nested spatial organization of brain tumor subregions, avoiding the use of multiple object proposals designed for independent objects.
2. We develop a two-stage 3D framework that combines subject-specific tumor localization with region-focused subregion segmentation.
3. We evaluate the contribution of the localization stage and the three decoder-fusion strategies through controlled comparisons between U-Net and ReU-Net and between the corresponding FusionNet and ReFusionNet variants.

## Methods

### Overview

The proposed end-to-end network primarily consists of two components: the localization module and the mask module. The corresponding structures are illustrated in **Figure 1**. First, the multi-modal MRI brain data is fed into the localization module, which estimates the position and spatial extent of the tumor-bearing region (**Figure 2**). Next, the corresponding region of interest is then extracted and passed to the mask module for subregion segmentation. Finally, the segmented tumor is mapped back to the original image space (**Figure 3**).

**Figure 1:**
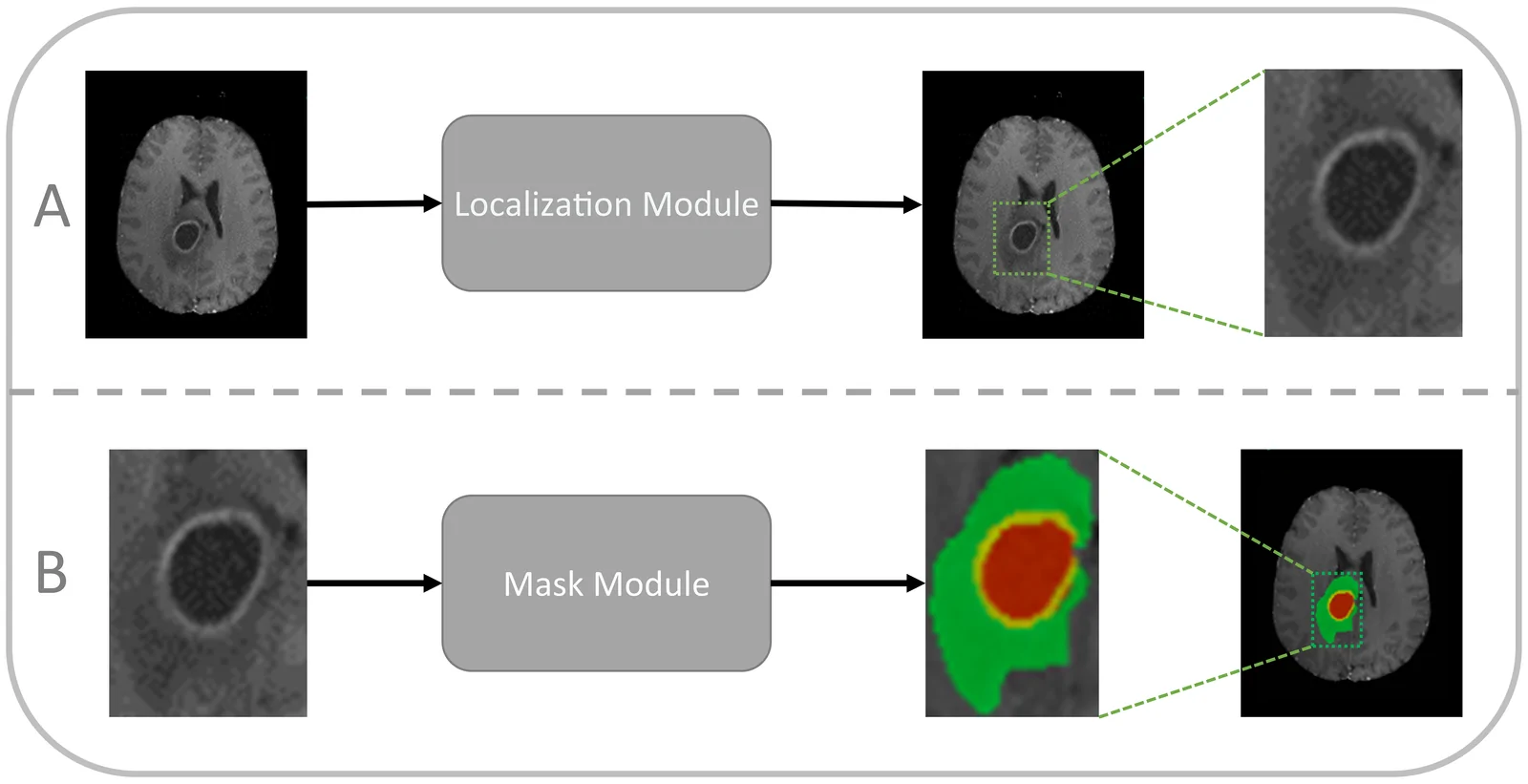
The flowchart of ReFusionNet. Note: **(A)** Localization stage: Multi-modal MRI brain data is input into the localization module, which identifies and outputs the tumor’s location and contour; **(B)** Mask stage: Based on the detected location and contour, the tumor is cropped from the original MRI data and passed into the mask module, which produces the segmentation results.

**Figure 2:**
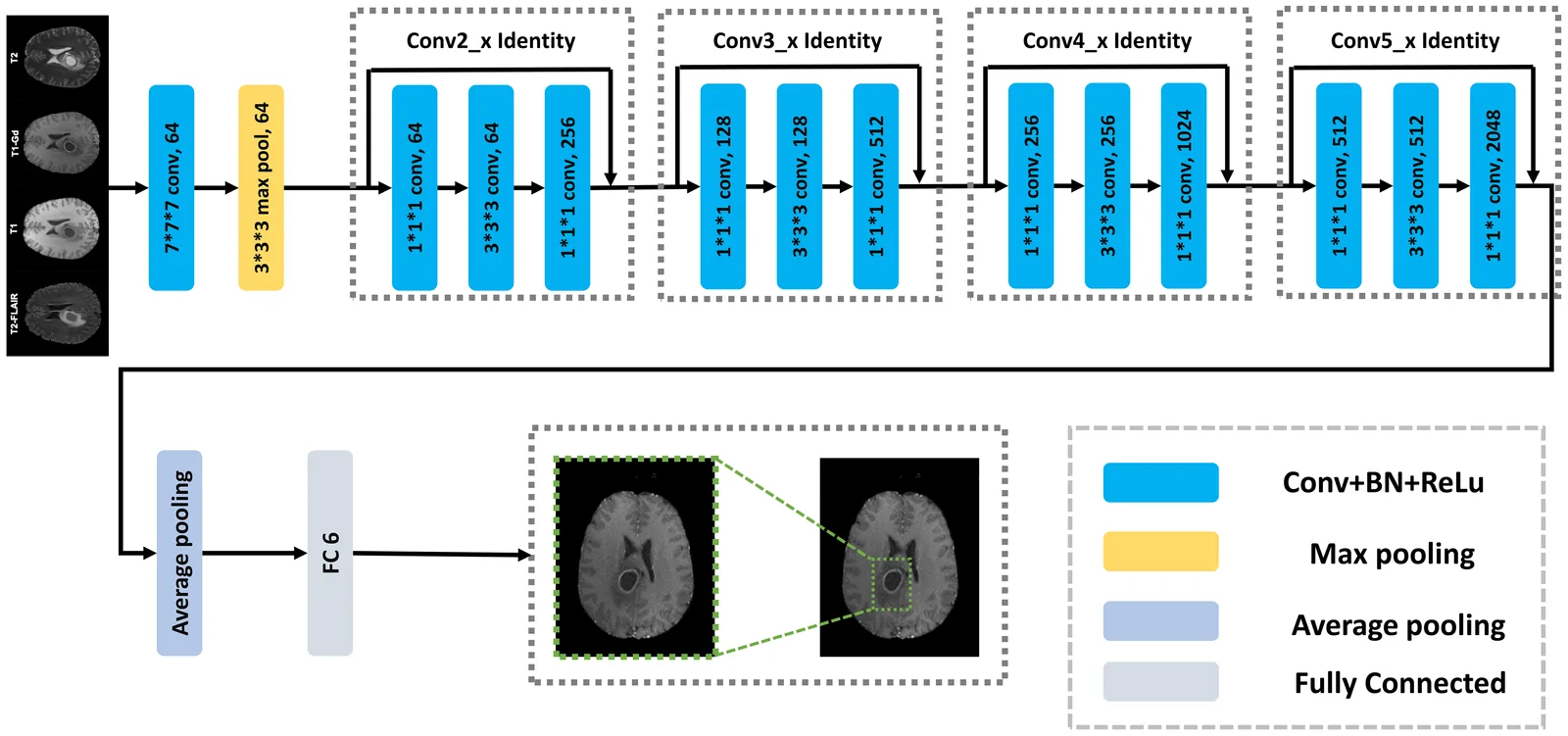
The network structure of localization module. Note: We employ a 3D ResNet101 as the backbone to detect the location and size of the tumors. The output layer (FC6) generates six parameters that are used to dynamically adjust the proposals for tumor detection.

**Figure 3:**
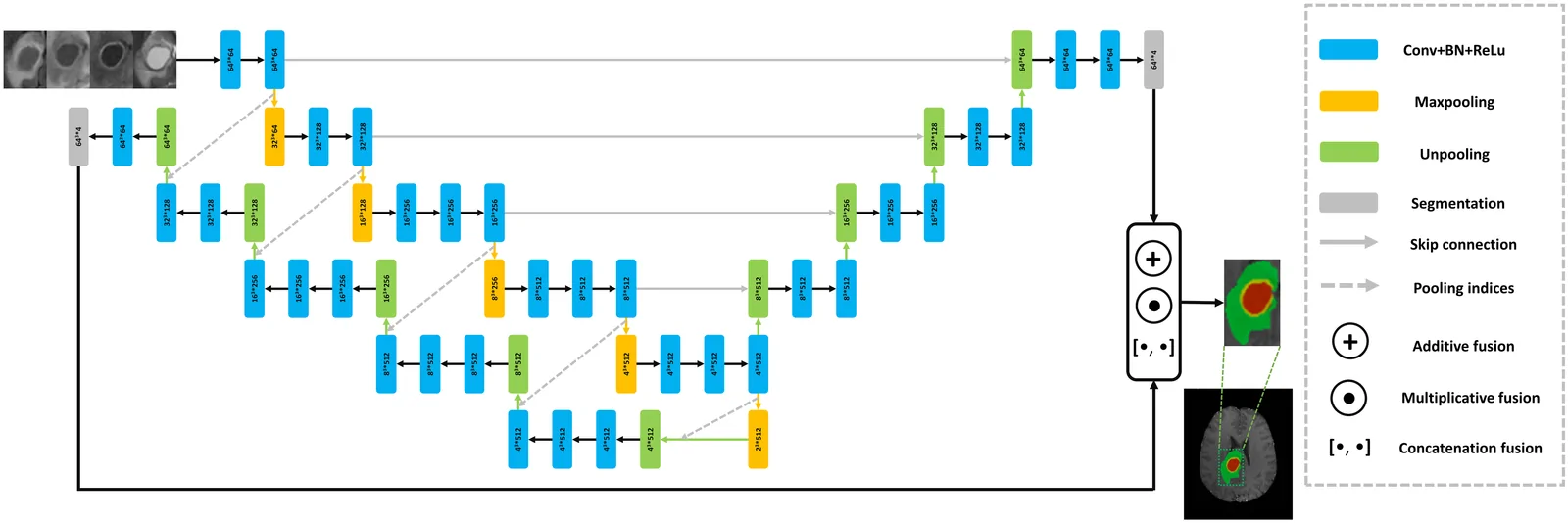
The network structure of mask module. Note: We implemented a FusionNet, which combines the architectures of 3D U-Net and 3D SegNet, sharing a common encoder to enhance feature extraction and improve segmentation performance.

### Tumor localization and proposal generation

The localization module identifies the tumor-containing region and generates a proposal box for the subsequent segmentation module. Unlike conventional object detection systems, our framework does not generate a separate proposal for each tumor sub-region. Instead, it uses a simplified proposal generation strategy tailored to brain tumor segmentation. Brain tumor sub-regions often have irregular shapes, ambiguous boundaries, and substantial spatial overlaps, making it difficult to represent them accurately using independent proposals generated by sliding-window methods, selective search, or region proposal networks. These conventional approaches may also introduce considerable computational overhead when applied to 3D MRI volumes. Therefore, the proposal box is initialized to cover the entire MRI volume, after which its location and size are adaptively adjusted for each subject according to the output of the localization module. The resulting proposal encompasses all tumor sub-regions and defines a tumor-focused region for subsequent segmentation. This design differs from conventional object detection in natural images, where spatially separate objects are usually represented by individual bounding boxes.

### Mask module

The localization module estimates the location and spatial extent of the tumor-containing region, which is then cropped from the original MRI volume. An analysis of the ground-truth bounding boxes in the training set showed that most tumor-containing regions had dimensions of approximately between 60 and 70 voxels. Accordingly, each cropped region is resized to a fixed input size of 64 × 64 × 64 voxels before being fed into the mask module. The mask module then predicts the segmentation masks for the three tumor sub-regions. Finally, the segmentation results are restored to the original size based on the specific location and size of the tumors.

### Data collection

The MRI data for this study was sourced from two public datasets: the UPENN-GBM dataset and the BraTS 2021 dataset. The UPENN-GBM dataset comprises MRI scans from a cohort of 630 GBM patients at the University of Pennsylvania Health System (UPENN-GBM) (20). This dataset encompasses imaging, clinical, and radiomic data and can be accessed via the Cancer Imaging Archive (TCIA) at the National Cancer Institute (21, 22). All MRI scans were collected prior to surgical intervention using 3T MRI scanners and included T1, T2, ceT1, FLAIR sequences, along with their corresponding segmentation labels. A total of 611 patients with complete MRI data were included in this analysis.

The BraTS 2021 dataset contains routine clinically acquired multi-parametric MRI (mpMRI) scans of gliomas collected from multiple institutions (23–25). Ground truth annotations for the tumor sub-regions were created and approved by expert neuroradiologists for each subject in the training, validation, and testing datasets, allowing quantitative evaluation of predicted tumor segmentations. Due to validation, and testing datasets only including corresponding MRI scans without public ground truth annotations. However, since the ground truth annotations for the validation and testing datasets are not publicly available, only the 1251 samples from the training dataset were included in this analysis.

### Data preprocessing and partitioning

The MRI data from UPENN-GBM was used in the preprocessed form released by the data provider. Provider-level preprocessing was not repeated in this study. To match the network input dimensions, we applied zero-padding, increasing their dimensions from the original 240×240×155 voxels to 256×256×256 voxels. At the subject level, 500 cases were assigned to training, 31 to validation, and 80 to testing. To augment the training set further, we employed a variety of data augmentation techniques, including horizontal and vertical flips, as well as rotations at 90, 180, and 270 degrees, resulting in a total of 3,000 training samples.

For the MRI data from the BraTS 2021 training dataset, similar preprocessing operations were applied. The 1251 samples were divided into a training set of 1000 cases, with online augmentation techniques used to enhance the data, a validation set of 51 cases, and a testing set of 200 cases.

### Architecture and implementation details

As described above, the proposed end-to-end network consists of two primary components: the localization module and the mask module. The localization module uses 3D ResNet101 as its backbone. The mask module uses FusionNet, which contains a shared 13-layer convolutional encoder based on VGG16 and two decoder pathways derived from U-Net and SegNet. Additionally, the network features an optional joint output layer for the decoders, supporting additive (A), elementwise multiplicative (M), and concatenation (C) operations. The additive (A) operation sums up the outputs from the two decoders, combining their features. The elementwise multiplicative (M) operation multiplies the outputs of the decoders on a per-element basis, enhancing areas where both agree. The concatenation (C) operation merges the outputs along the channel dimension, preserving distinct features from both decoders for more comprehensive representation.

The loss function used in this network combines smooth L1 loss (Equation 1) and cross-entropy loss. Smooth L1 loss is commonly employed in tasks like object detection (Equation 2), as it balances sensitivity to both small and large errors. Cross-entropy loss, on the other hand, measures voxel-wise similarity between the predicted segmentation and the ground truth. The specific computation equations are as follows:

(1) $$L_{box}=\{\begin{array}{c} 0.5\times x^{2},\;\;\;|x|<1\\ |x|-0.5,\;\;otherwise \end{array}$$

Where *x* is the difference between the predicted and target values $\left(x=y_{pred}-y_{true}\right)$.

(2) $$L_{mask}=-\frac{1}{H\times W\times D}\sum_{h=1}^{H}\sum_{w=1}^{W}\sum_{d=1}^{D}\sum_{c=1}^{C}y_{h,w,d,c}\log({\hat{y}}_{h,w,d,c})$$

Where $y_{h,w,d,c}$ represents the true label for the pixel at position (h,w,d) for class *c*, and ${\hat{y}}_{h,w,d,c}$ is the predicted probability for voxel (h,w,d) belonging to class *c*, as given by the model. The loss is averaged over all voxels to obtain a single scalar value representing the loss for the entire image.

(3) $$L_{total}=L_{box}+L_{mask}$$

Where $L_{box}$ represents the smooth L1 loss, $L_{mask}$ is the cross-entropy loss, and $L_{total}$ is the total loss for the entire model (Equation 3).

In this study, we utilized four standard MRI modalities along with their corresponding expert-annotated segmentation maps to train the network. Both training and testing were performed on an 80 GB NVIDIA A100 Tensor Core GPU using the Torch backend. The model’s initial parameters were set using PyTorch’s default initialization, and network parameters were updated with the stochastic gradient descent (SGD) optimization algorithm. The learning rate was set to 0.01 and decayed exponentially with a factor of 0.8. Training was carried out over 20 epochs with a batch size of 10.

### Performance metrics

In this study, the segmentation performance of models was evaluated by recall, precision, Dice score and Hausdorff distance (HD95). Recall measures the ability of the model to correctly identify positive instances (Equation 4).

(4) $$Recall=\frac{TP}{TP+FN}$$

Precision measures the accuracy of the positive predictions (Equation 5).

(5) $$Precision=\frac{TP}{TP+FP}$$

Dice Score is the harmonic mean of precision and recall, providing a single metric to evaluate the balance between the two (Equation 6).

(6) $$Dice=\frac{2\times TP}{TP+FN+TP+FP}$$

True Positive (TP) means the number of instances correctly predicted as positive; false positive (FP) means the number of instances incorrectly predicted as positive; true negative (TN) means the number of instances correctly predicted as negative; false negative (FN) means the number of instances incorrectly predicted as negative.

The Hausdorff distance (HD) is a metric used to measures the maximum discrepancy between predicted and ground truth segmentations in medical image segmentation (Equation 7). The HD95 refines this by taking the 95th percentile of those distances, which helps avoid the influence of outliers or noise.

(7) $$HD(T,P)=\text{max}\{\underset{t\in T}{\sup}\underset{p\in P}{\inf}d(t,p),\underset{p\in P}{\sup}\underset{t\in T}{\inf}d(t,p)\}$$

Where *t* and *p* represent the points on the ground truth regions *T* and the predicted regions *P*, respectively. d(t,p) denotes the distance between point *t* and *p*.

Regarding the tumor sub-regions, the definitions align with convention for comparison with other studies. The whole tumor refers to the combined areas of the necrotic core, enhancing tumor, and peritumoral edema regions. The tumor core refers to the combined areas of the necrotic core and enhancing tumor regions. The enhancing tumor still refers specifically to the enhancing tumor areas.

### Statistical summary

Recall, precision, Dice score, and HD95 were calculated separately for each subject in the corresponding test set. For each model, the subject-level results are summarized as the mean and 95% confidence interval (CI) across test subjects. All models compared within the same experiment were evaluated using the same data partition.

## Results

In this work, we conducted three comparative experiments. The first experiment compared full-volume and localization-guided models and evaluated the decoder-fusion strategies. The second experiment evaluated the segmentation results using various combinations of MRI modalities. The third experiment compared the performance of the proposed methods with several state-of-the-art models on the BraTS 2021 dataset.

### Effects of localization-guided ROI segmentation and decoder fusion

We compared the full-volume models (3D U-Net and 3D FusionNet) with their localization-guided ROI counterparts (3D ReU-Net and 3D ReFusionNet) using FLAIR, T1, ceT1, and T2 MRI modalities (**Table 1**). The localization-guided models generally maintained comparable Dice scores for the whole tumor while achieving higher precision and lower HD95 values. ReU-Net and U-Net both obtained a whole-tumor Dice score of 0.89, whereas ReFusionNet-A and FusionNet-A both achieved 0.90. However, the Dice scores of the localization-guided models were lower in some tumor-core and enhancing-tumor comparisons. For example, ReFusionNet-A achieved Dice scores of 0.81 and 0.77 for the tumor core and enhancing tumor, respectively, compared with 0.84 for both regions using FusionNet-A.

**Table 1:** Summary of performance metrics for ablation methods.

Methods	Recall			Precision			Dice			HD95		
	WT	TC	ET	WT	TC	ET	WT	TC	ET	WT	TC	ET
U-Net	0.87 [0.85, 0.89]	0.69 [0.64, 0.74]	0.00 [0.00, 0.00]	0.93 [0.92, 0.94]	0.86 [0.83, 0.89]	0.12 [0.06, 0.18]	0.89 [0.87, 0.91]	0.73 [0.69, 0.77]	0.00 [0.00, 0.00]	29.97 [23.88, 36.06]	23.72 [18.66, 28.78]	27.19 [21.39, 32.99]
FusionNet-A	**0.89 [0.87, 0.91]**	0.84 [0.81, 0.87]	**0.87 [0.85, 0.89]**	0.92 [0.90, 0.94]	0.87 [0.84, 0.90]	0.84 [0.82, 0.86]	**0.90 [0.88, 0.92]**	0.84 [0.82, 0.86]	**0.84 [0.82, 0.86]**	30.41 [24.07, 36.75]	19.13 [15.41, 22.85]	16.61[13.24, 19.98]
FusionNet-M	0.89 [0.86, 0.92]	0.72 [0.68, 0.76]	0.82 [0.78, 0.86]	0.91 [0.89, 0.93]	0.91 [0.89, 0.93]	0.79 [0.77, 0.81]	0.89 [0.86, 0.92]	0.79 [0.76, 0.82]	0.79 [0.77, 0.81]	46.79 [40.29, 53.29]	20.75 [16.95, 24.55]	20.68 [16.88, 24.48]
FusionNet-C	0.88 [0.86, 0.90]	**0.88 [0.86, 0.90]**	0.82 [0.79, 0.85]	0.91 [0.90, 0.92]	0.85 [0.82, 0.88]	0.77 [0.75, 0.79]	0.90 [0.89, 0.91]	**0.86 [0.84, 0.88]**	0.78 [0.76, 0.80]	25.79 [20.41, 31.17]	**15.18 [11.29, 19.07]**	14.08 [10.35, 17.81]
ReU-Net	0.84 [0.82, 0.86]	0.70 [0.68, 0.72]	0.62 [0.60, 0.64]	**0.97 [0.96, 0.98]**	0.91 [0.89, 0.93]	0.95 [0.93, 0.97]	0.89 [0.87, 0.91]	0.78 [0.76, 0.80]	0.74 [0.72, 0.76]	12.60 [9.84, 15.36]	21.64 [18.95, 24.33]	11.10 [8.77, 13.43]
ReFusionNet-A	0.85 [0.83, 0.87]	0.72 [0.70, 0.74]	0.66 [0.64, 0.68]	0.96 [0.95, 0.97]	**0.93 [0.91, 0.95]**	0.95 [0.94, 0.96]	0.90 [0.89, 0.91]	0.81 [0.80, 0.82]	0.77 [0.76, 0.78]	**11.68 [9.63, 13.73]**	21.53 [18.91, 24.15]	10.49 [8.30, 12.68]
ReFusionNet-M	0.85 [0.83, 0.87]	0.67 [0.65, 0.69]	0.57 [0.54, 0.60]	0.96 [0.95, 0.97]	0.87 [0.85, 0.89]	0.96 [0.94, 0.98]	0.89 [0.87, 0.91]	0.75 [0.73, 0.77]	0.70 [0.68, 0.72]	13.78 [10.95, 16.61]	20.68 [18.07, 23.29]	10.18 [8.03, 12.33]
ReFusionNet-C	0.85 [0.83, 0.87]	0.69 [0.67, 0.71]	0.59 [0.57, 0.61]	0.96 [0.95, 0.97]	0.93 [0.91, 0.95]	**0.96 [0.95, 0.97]**	0.90 [0.88, 0.92]	0.79 [0.77, 0.81]	0.73 [0.71, 0.75]	12.77 [9.98, 15.56]	20.09 [17.54, 22.64]	**10.08 [8.06, 12.10]**

The evaluated metrics are presented as mean (95% CI). WT, Whole tumor areas; TC, Tumor core areas; ET, Enhancing Tumor areas; HD, Hausdorff distance.Bold values indicate the best result in each column.

The localization-guided models generally showed higher precision but lower recall than their full-volume counterparts. For the whole tumor, ReU-Net and the three ReFusionNet variants achieved precision values of 0.96–0.97, compared with 0.91–0.92 for the FusionNet variants. A similar pattern was observed for the enhancing tumor, for which the localization-guided models achieved precision values of 0.95–0.96, compared with 0.77–0.84 for the FusionNet variants. In contrast, their recall values were generally lower, particularly for the tumor core and enhancing tumor. For example, ReFusionNet-A achieved an enhancing-tumor recall of 0.66, whereas FusionNet-A achieved 0.87.

The clearest improvement associated with localization-guided ROI segmentation was observed for HD95. For the whole tumor, ReU-Net and the ReFusionNet variants achieved HD95 values of 11.68–13.78, whereas their full-volume counterparts achieved values of 25.79–46.79. For the enhancing tumor, the corresponding HD95 values were 10.08–11.10 and 14.08–27.19, respectively. This pattern was not consistently observed for the tumor core, for which FusionNet-C achieved the lowest HD95 of 15.18. Overall, localization-guided ROI segmentation improved precision and boundary accuracy for the whole tumor and enhancing tumor, but these improvements were accompanied by lower recall and Dice scores in some comparisons.

### Segmentation performance with different combinations of MRI modalities

In the following section, we evaluated the effectiveness of segmentation results from various combinations of MRI modalities based on ReFusionNet-A (**Table 2**). The combination of all four modalities (FLAIR, T1, ceT1, and T2) achieved the best overall performance. The corresponding Dice scores were 0.90 for the whole tumor, 0.81 for the tumor core, and 0.77 for the enhancing tumor. The recall and precision values were 0.85 and 0.96 for the whole tumor, 0.72 and 0.93 for the tumor core, and 0.66 and 0.95 for the enhancing tumor, respectively.

**Table 2:** Summary of performance metrics for different combinations of MRI modalities based on ReFusionNet-A.

Modalities	Recall			Precision			Dice		
	WT	TC	ET	WT	TC	ET	WT	TC	ET
FLAIR/T1/T1c/T2	0.85 [0.83, 0.87]	**0.72 [0.70, 0.74]**	**0.66 [0.64, 0.68]**	**0.96 [0.95, 0.97]**	**0.93 [0.91, 0.95]**	0.95 [0.94, 0.96]	**0.90 [0.89, 0.91]**	**0.81 [0.80, 0.82]**	**0.77 [0.76, 0.78]**
FLAIR/T1/T1c	**0.86 [0.84, 0.88]**	0.64 [0.62, 0.66]	0.56 [0.54, 0.58]	0.94 [0.93, 0.95]	0.90 [0.88, 0.92]	**0.97 [0.96, 0.98]**	0.90 [0.89, 0.91]	0.74 [0.72, 0.76]	0.70 [0.68, 0.72]
FLAIR/T1/T2	0.85 [0.83, 0.87]	0.32 [0.26, 0.38]	0.09 [0.05, 0.13]	0.95 [0.94, 0.96]	0.69 [0.64, 0.74]	0.61 [0.53, 0.69]	0.88 [0.86, 0.90]	0.36 [0.31, 0.41]	0.11 [0.08, 0.14]
FLAIR/T1c/T2	0.81 [0.77, 0.85]	0.54 [0.50, 0.58]	0.40 [0.36, 0.44]	0.91 [0.88, 0.94]	0.85 [0.80, 0.90]	0.92 [0.87, 0.97]	0.84 [0.81, 0.87]	0.62 [0.58, 0.66]	0.52 [0.48, 0.56]
T1/T1c/T2	0.50 [0.45, 0.55]	0.53 [0.49, 0.57]	0.45 [0.41, 0.49]	0.84 [0.78, 0.90]	0.72 [0.66, 0.78]	0.90 [0.85, 0.95]	0.61 [0.56, 0.66]	0.59 [0.54, 0.64]	0.57 [0.52, 0.62]
FLAIR/T1	**0.83 [0.80, 0.86]**	0.28 [0.23, 0.33]	0.04 [0.02, 0.06]	**0.93 [0.91, 0.95]**	0.63 [0.57, 0.69]	0.34 [0.28, 0.40]	**0.87 [0.84, 0.90]**	0.33 [0.28, 0.38]	0.05 [0.03, 0.07]
FLAIR/T1c	0.76 [0.72, 0.80]	**0.43 [0.39, 0.47]**	0.20 [0.17, 0.23]	0.82 [0.78, 0.86]	**0.64 [0.59, 0.69]**	**0.88 [0.82, 0.94]**	0.76 [0.72, 0.80]	**0.47 [0.43, 0.51]**	0.27 [0.23, 0.31]
FLAIR/T2	0.79 [0.75, 0.83]	0.30 [0.24, 0.36]	0.05 [0.02, 0.08]	0.90 [0.87, 0.93]	0.59 [0.53, 0.65]	0.51 [0.43, 0.59]	0.83 [0.79, 0.87]	0.33 [0.28, 0.38]	0.06 [0.03, 0.09]
T1/T1c	0.48 [0.41, 0.55]	0.32 [0.27, 0.37]	**0.27 [0.22, 0.32]**	0.26 [0.21, 0.31]	0.42 [0.36, 0.48]	0.81 [0.74, 0.88]	0.27 [0.23, 0.31]	0.32 [0.27, 0.37]	**0.37 [0.31, 0.43]**
T1/T2	0.44 [0.37, 0.51]	0.27 [0.21, 0.33]	0.01 [0.00, 0.02]	0.69 [0.62, 0.76]	0.37 [0.30, 0.44]	0.13 [0.08, 0.18]	0.51 [0.44, 0.58]	0.26 [0.21, 0.31]	0.01 [0.00, 0.02]
T1c/T2	0.42 [0.37, 0.47]	0.32 [0.28, 0.36]	0.22 [0.18, 0.26]	0.61 [0.53, 0.69]	0.50 [0.43, 0.57]	0.74 [0.66, 0.82]	0.45 [0.39, 0.51]	0.35 [0.30, 0.40]	0.30 [0.26, 0.34]
FLAIR	**0.61 [0.56, 0.66]**	**0.29 [0.24, 0.34]**	**0.06 [0.04, 0.08]**	**0.77 [0.72, 0.82]**	**0.50 [0.44, 0.56]**	**0.42 [0.36, 0.48]**	**0.66 [0.61, 0.71]**	**0.32 [0.27, 0.37]**	**0.07 [0.05, 0.09]**
T1	0.02 [0.01, 0.03]	0.01 [0.01, 0.01]	0.00 [0.00, 0.00]	0.20 [0.13, 0.27]	0.11 [0.05, 0.17]	0.04 [0.00, 0.08]	0.03 [0.01, 0.05]	0.01 [0.00, 0.02]	0.00 [0.00, 0.00]
T1c	0.06 [0.04, 0.08]	0.01 [0.00, 0.02]	0.01 [0.01, 0.01]	0.13 [0.08, 0.18]	0.15 [0.09, 0.21]	0.25 [0.16, 0.34]	0.06 [0.04, 0.08]	0.02 [0.01, 0.03]	0.02 [0.01, 0.03]
T2	0.33 [0.28, 0.38]	0.16 [0.11, 0.21]	0.01 [0.01, 0.01]	0.63 [0.55, 0.71]	0.34 [0.27, 0.41]	0.09 [0.05, 0.13]	0.39 [0.33, 0.45]	0.17 [0.12, 0.22]	0.01 [0.00, 0.02]

The evaluated metrics are presented as mean (95% CI). WT, Whole tumor areas; TC, Tumor core areas; ET, Enhancing Tumor areas.Bold values indicate the best result in each column.

The contribution of each modality varied across the tumor sub-regions. Among the individual modalities, FLAIR achieved the best whole-tumor segmentation, with a Dice score of 0.66, but showed limited performance for the tumor core and enhancing tumor, with Dice scores of 0.32 and 0.07, respectively. Modality combinations containing ceT1 generally performed better for the tumor core and enhancing tumor. For example, the T1–ceT1 combination achieved an enhancing-tumor recall of 0.27 and a Dice score of 0.37, whereas the corresponding values for FLAIR–T1 were 0.04 and 0.05. These results indicate that FLAIR contributed primarily to whole-tumor delineation, whereas ceT1 provided important information for identifying the tumor core and enhancing tumor.

### Visualization of brain tumor segmentation

**Figure 4** presents the segmentation results of the different models for a representative subject in the axial plane. From left to right, the images showcase the original T2 image, the ground truth, and the segmentation results from the corresponding models. Red indicates the necrotic core regions, yellow denotes the enhancing tumor areas, and green represents the peritumoral edema regions. In this example, the predictions generated by ReU-Net, ReFusionNet-A, and ReFusionNet-C showed closer visual agreement with the ground-truth annotation. U-Net missed part of the enhancing tumor, whereas FusionNet-M showed inaccurate delineation of the necrotic core. Some misclassifications between the necrotic core and peritumoral edema was also observed. Specifically, FusionNet-A and ReFusionNet-M classified portions of the peritumoral edema as necrotic core, whereas FusionNet-C classified part of the necrotic core as peritumoral edema. Similar visual patterns were observed in the coronal and sagittal views shown in **Figures 5**, **6**.

**Figure 4:**
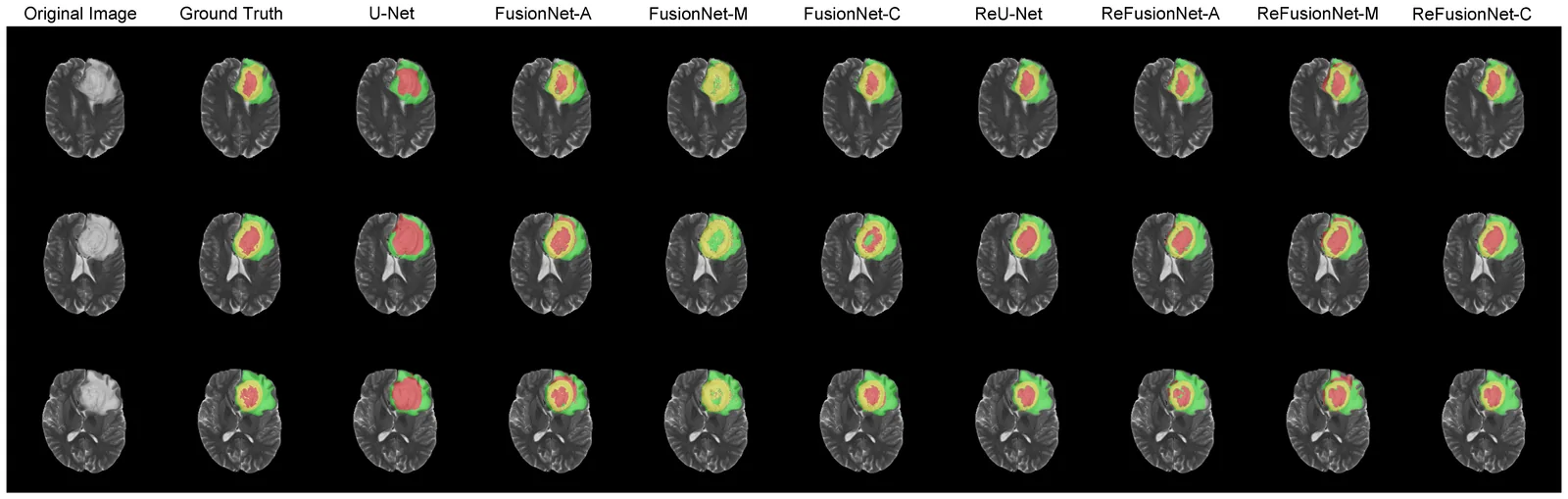
Segmentation results from a randomly selected subject in the axial plane. The first column displays the original images, the second column shows the ground truths, and the remaining columns present the predicted segmentation results from different models. Each row corresponds to different slices of T2 MRI images. In the segmentation results, red indicates necrotic core regions, yellow represents enhancing tumor regions, and green denotes peritumoral edema regions.

**Figure 5:**
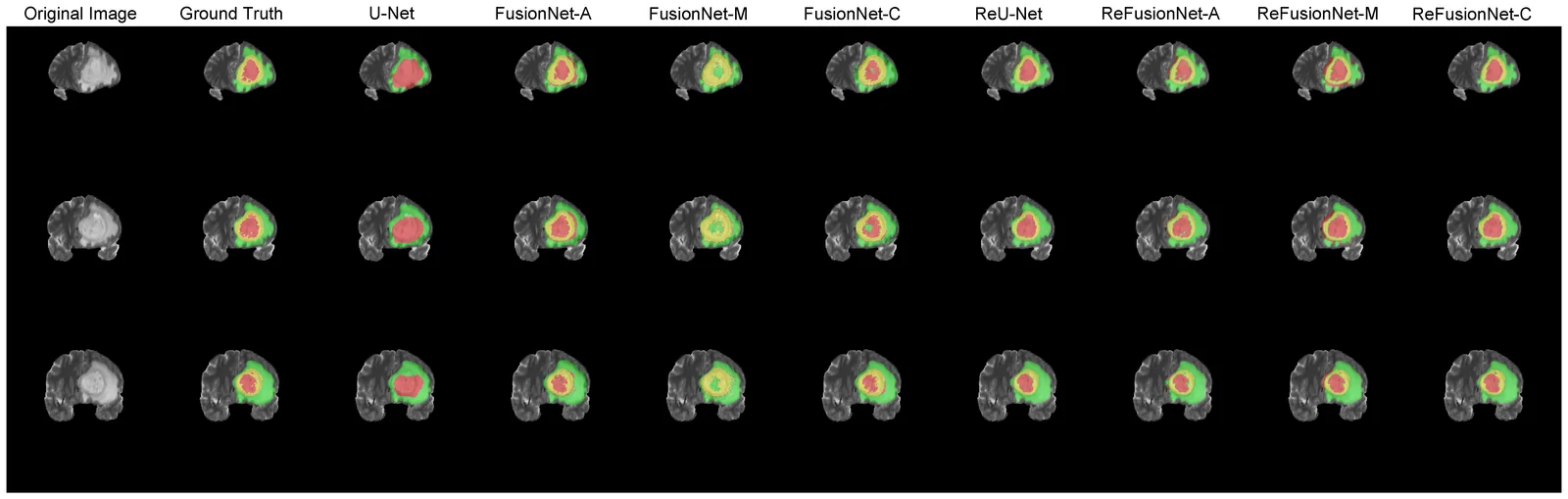
Segmentation results from a randomly selected subject in the coronal plane. The first column displays the original images, the second column shows the ground truths, and the remaining columns present the predicted segmentation results from different models. Each row corresponds to different slices of T2 MRI images. In the segmentation results, red indicates necrotic core regions, yellow represents enhancing tumor regions, and green denotes peritumoral edema regions.

**Figure 6:**
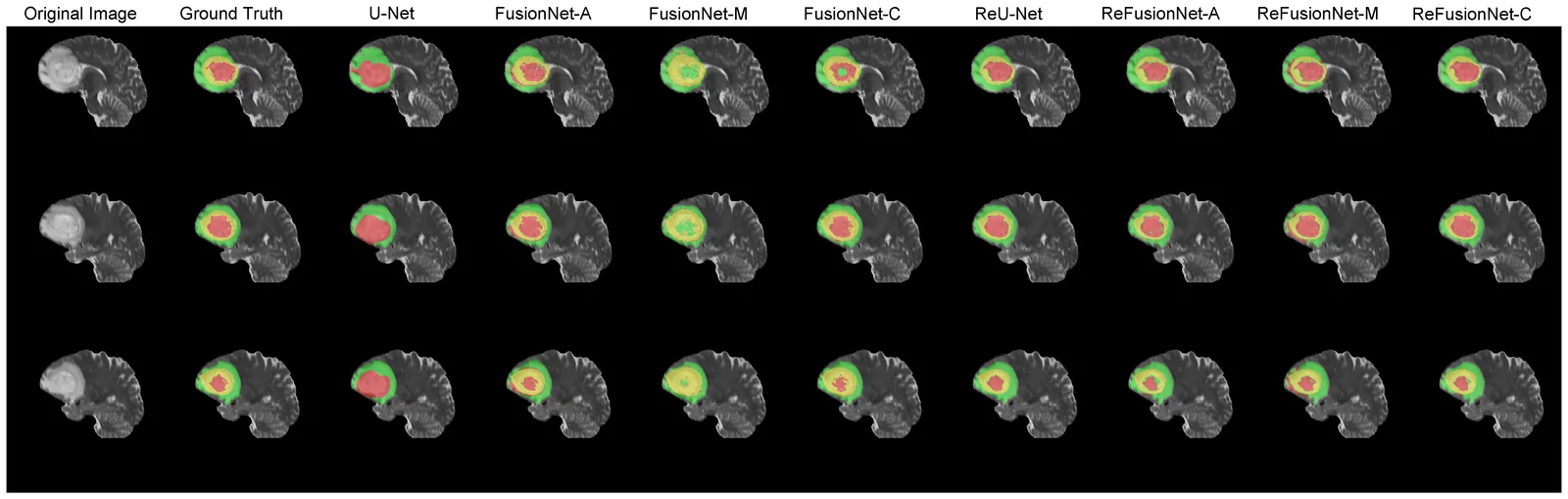
Segmentation results from a randomly selected subject in the sagittal plane. The first column displays the original images, the second column shows the ground truths, and the remaining columns present the predicted segmentation results from different models. Each row corresponds to different slices of T2 MRI images. In the segmentation results, red indicates necrotic core regions, yellow represents enhancing tumor regions, and green denotes peritumoral edema regions.

### Segmentation performance with different methods

Additionally, we conducted comparative experiments based on ReFusionNet-C alongside several state-of-the-art models on the BraTS 2021 dataset, with the results presented in **Table 3**. In this section, we primarily focus on the Dice score and HD95 metrics to evaluate the segmentation performance of the different models. The models included in this comparison are V-Net, U-Net, Attention U-Net, Res U-Net and Dense-Attention 3D U-Net -DAF (2) [Dense-Attention-Fusion-Focal, DAF (2)] (26–30).

**Table 3:** Summary of performance metrics for different methods.

Methods	Dice			HD95		
	WT	TC	ET	WT	TC	ET
V-Net	0.85	0.68	0.61	9.45	28.94	6.06
U-Net	0.87	0.71	0.65	5.94	27.10	3.41
Attention U-Net	0.89	0.76	0.70	**4.80**	**20.42**	**2.32**
Res U-Net	0.89	0.78	0.72	6.42	23.37	4.26
DAF^2^	0.90	0.81	0.76	5.72	22.22	3.34
ReFusionNet-C	**0.90**	**0.83**	**0.80**	8.73	24.53	5.61

The evaluated metrics are presented as mean. WT, Whole tumor areas; TC, Tumor core areas; ET, Enhancing Tumor areas; HD, Hausdorff distance.Bold values indicate the best result in each column.

ReFusionNet-C achieved Dice scores of 0.90, 0.83, and 0.80 for the whole tumor, tumor core, and enhancing tumor, respectively. The corresponding highest values reported by the other methods were 0.90, 0.81, and 0.76. Thus, ReFusionNet-C achieved competitive Dice performance, particularly for the tumor core and enhancing tumor. However, it did not achieve the lowest HD95 value. ReFusionNet-C obtained HD95 values of 8.73, 24.53, and 5.61 for the three regions, whereas Attention U-Net achieved 4.80, 20.42, and 2.32, respectively. These results indicate that ReFusionNet-C performed competitively in terms of regional overlap, while its boundary-distance performance remained less favorable than that of Attention U-Net.

## Discussion

Brain tumors are among the most life-threatening human diseases, making accurate diagnosis and assessment essential. MRI provides detailed soft-tissue information, and accurate tumor segmentation supports diagnosis, treatment planning, and disease monitoring in patients with glioblastoma and other brain tumors (31). This process enables clinicians to visualize tumor boundaries, assess size and type, and guide surgical interventions. However, manual segmentation by radiologists is time-consuming, labor-intensive, and susceptible to interobserver variability (32). Deep learning-based methods have therefore been increasingly investigated to automate brain tumor segmentation.

Accurate segmentation of the necrotic core (NCR), peritumoral edema (ED), and enhancing tumor (ET) provides clinically relevant information about tumor composition and spatial extent (33). Delineation of these sub-regions may support treatment planning, evaluation of treatment response, and assessment of disease progression or recurrence (34). Reliable sub-region segmentation may therefore contribute to more individualized management of patients with brain tumors.

In this study, we developed a two-stage, localization-guided framework for 3D brain tumor segmentation. The framework first identifies a tumor-containing ROI and then segments the three tumor sub-regions within the localized volume. The results indicate that localization-guided ROI segmentation did not uniformly improve all evaluation metrics. Compared with the corresponding full-volume models, ReU-Net and ReFusionNet generally achieved higher precision and lower HD95 values for the whole tumor and enhancing tumor. However, lower recall and Dice scores were observed in some comparisons involving the tumor core and enhancing tumor. Therefore, the principal effect of the proposed strategy was a change in the balance between false-positive suppression, boundary accuracy, and tumor-voxel sensitivity, rather than an overall improvement across all metrics.

The higher precision of the localization-guided models may result from restricting segmentation to a tumor-containing ROI. By reducing the amount of irrelevant background processed by the mask module, this strategy may limit false-positive predictions outside the tumor region. The lower HD95 values observed for the whole tumor and enhancing tumor also suggest fewer large boundary deviations. However, the effectiveness of this strategy depends on the accuracy of the localization module. An inaccurately estimated proposal box may exclude peripheral tumor voxels or affect their representation during resizing, which may partly explain the lower recall observed for some tumor sub-regions. This dependence on localization accuracy is an important characteristic and potential limitation of the proposed framework.

We also investigated the effects of different MRI modality combinations. The combination of FLAIR, T1, ceT1, and T2 achieved the best overall segmentation performance, indicating that the different sequences provide complementary information. Among the individual modalities, FLAIR achieved the highest whole-tumor segmentation performance. This may be related to its sensitivity to peritumoral edema, which appears as a region of high signal intensity. In contrast, modality combinations containing ceT1 generally performed better for the tumor core and enhancing tumor because contrast enhancement improves the visibility of active tumor tissue. These findings support the use of multimodal MRI for distinguishing tumor sub-regions with different imaging characteristics.

ReFusionNet-C achieved competitive Dice scores compared with the representative methods reported for the BraTS 2021 dataset. However, it did not achieve the lowest HD95 value, particularly when compared with Attention U-Net. This difference indicates that regional overlap and boundary-distance metrics reflect different aspects of segmentation performance. A high Dice score does not necessarily correspond to the lowest boundary error. In addition, the results of the comparison methods were obtained from their respective publications and may have been influenced by differences in data partitioning, preprocessing, and implementation settings. Therefore, **Table 3** provides a contextual comparison rather than a controlled demonstration of superiority.

Compared with conventional proposal generation methods, the proposed localization module generates a single adaptive proposal box that encompasses all tumor sub-regions. The proposal is initialized to cover the entire MRI volume, and its location and size are then adjusted for each subject. The resulting ROI reduces the spatial extent processed by the mask module and allows the segmentation stage to focus on the tumor-containing region. Nevertheless, computational efficiency was not quantitatively evaluated in terms of model parameters, floating-point operations, GPU memory consumption, or inference time. Consequently, the present results do not establish that the complete framework is computationally more efficient than existing methods.

Although the results are encouraging, further evaluation is warranted. The present study used two publicly available datasets, and validation using an independent clinical cohort would provide additional evidence of generalizability. More detailed component analyses and quantitative evaluation of computational requirements may also help characterize the framework more comprehensively. Future work will therefore focus on external validation and further refinement of the localization and mask modules.

## Conclusion

This study presents a localization-guided framework for automated 3D brain tumor segmentation. The framework first estimates the location and spatial extent of the tumor-containing region and then segments the tumor sub-regions within the resulting ROI. The results indicate that this strategy can improve precision and boundary accuracy for the whole tumor and enhancing tumor, although the improvements were not consistent across all regions and evaluation metrics. Further refinement of the localization module may provide more accurate ROI estimation and improve subsequent segmentation performance.

## Data Availability

The datasets analyzed for this study are publicly available in The Cancer Imaging Archive (TCIA). The names of the repository/repositories and accession number(s) can be found in the article/supplementary material.
